# CX691, as an AMPA receptor positive modulator, improves the learning and memory in a rat model of Alzheimer’s disease

**DOI:** 10.22038/IJBMS.2018.28544.6934

**Published:** 2018-07

**Authors:** Nazanin Mozafari, Ali Shamsizadeh, Iman Fatemi, Mohammad Allahtavakoli, Amir Moghadam-Ahmadi, Elham Kaviani, Ayat Kaeidi

**Affiliations:** 1 Physiology-Pharmacology Research Center, Rafsanjan University of Medical Sciences, Rafsanjan, Iran; 2 Department of Physiology and Pharmacology, School of Medicine, Rafsanjan University of Medical Sciences, Rafsanjan, Iran; 3 School of Medicine and Non-Communicable Diseases Research Center, Rafsanjan University of Medical Sciences, Rafsanjan, Iran

**Keywords:** Alzheimer’s disease, AMPA receptors, BDNF, CX691, Memory, Rat

## Abstract

**Objective(s):**

Growing evidence suggests that dysfunction of the glutamatergic system and α-amino-3-hydroxy-5-methyl-4-isoazolepropionic acid (AMPA) receptors are involved in pathology of Alzheimer’s disease (AD). Because AMPA receptors play a key role in plasticity synaptic regulation, positive modulation of these receptors may rescue the cognitive deficits in the AD. The aim of this study was to explore the effect of CX691, a specific positive allosteric modulator of the AMPA-type glutamate receptors (Ampakine), on spatial learning and memory in a rat model of AD.

**Materials and Methods:**

For induction of AD, amyloid-beta 1-42 (Aβ1-42) was microinjected into the hippocampus of male Wistar rats (250-300 g). The Morris water maze (MWM) test was used to evaluate the effect of CX691 (0.03 and 0.3 mg/kg, twice a day for 10 days, orally) on spatial learning and memory of rats. In order to evaluate the protein expression of brain-derived neurotrophic factor (BDNF) in hippocampus tissue, ELISA test was used.

**Results:**

The obtained data showed that treatment with CX691 (0.3 mg/kg) improves the impairment of spatial learning and memory in AD rats. Also, treatment with CX691 (0.3 mg/kg), increased the BDNF protein level in hippocampus tissue of AD rats compared to non-treated animals.

**Conclusion:**

The CX691 can improve the BDNF protein expression as well as spatial performance of learning and memory in AD rats.

## Introduction

Alzheimer’s disease (AD) is a progressive and irreversible neurodegenerative disorder that occurs gradually and leads to memory loss, personality changes, unusual behavior and deficit in thinking abilities (1). The most common reason of dementia in the elderly peoples is AD (2). 

One of the first signs of AD is cognitive decline, which has been shown to be associated with synapse loss in animal AD models and AD human brain, as well as decreased α-amino-3-hydroxy-5-methyl-4-isoazolepropionic acid (AMPA) receptor-mediated synaptic transmission in animal AD models (3–5)genetic, animal model, and biochemical studies has indicated that the accumulation of amyloid-beta (Aβ). 

Enhancement of AMPA receptor activity is believed to upregulate glutamatergic function through both AMPA and N-Methyl-D-aspartic acid (NMDA) receptors (6). Facilitation of glutamatergic neurotransmission has been shown to result in long-term potentiation (LTP), a synaptic plasticity form that is suggested to be very important for learning and memory, and a crucial role of AMPA receptors in this process has clearly been established (7). Additionally, it has been shown that expression of brain-drive nerve factor (BDNF) at both mRNA and protein levels is decreased in specific brain areas of postmortem samples of AD, particularly in the hippocampus (8,9). Moreover, BDNF deficiency may result in amyloid beta (Aβ)-associated neurotoxicity and atrophy of dendrite (10).

Reinstating the loss of glutamatergic function in various psychiatric disorders, such as schizophrenia, attention-deficit hyperactivity disorder, depression and anxiety can be achieved by alterations in AMPA receptor function either through direct agonism or positive modulation (11). 

There are numerous classes of potent AMPA positive modulators (Ampakines) based on structure. These agents potentiate the AMPA receptor function and increase glutamatergic synaptic transmission.

The effects of ampakines on memory have been studied in both animals and human. In addition, there is evidence to suggest that they may enhance memory function (12,13). Furthermore, some recently developed ampakines upregulate the BDNF mRNA expression (14). Given that BDNF enhances and regulates the induction of synaptic plasticity, ampakines are ideal for the cognitive deficits treatment (15).

CX-691 or farampator (5-(1-piperidinylcarbonyl)-2,1,3-benzoxadiazole), is a specific positive allosteric modulator of the AMPA-type glutamate receptors (16). CX-691 has been developed for improvement of cognitive impairment and schizophrenia negative symptoms (17). CX691, at micromolar concentrations, potentiates AMPA receptor-mediated responses in a number of *in vitro* assay systems and improves hippocampal long-term potentiation, suggesting that CX691 may have cognitive enhancing effects with no serious or severe adverse events (13). Furthermore, we recently showed that administration of CX691 to Aβ-treated rats can improve working memory in these animals using Y-maze spontaneous alternation test (18). On the basis of these results, ampakines were thought to be potential drugs that could increase cognition in various neurological disorders (19). Thus, we explored the effect of CX691 on spatial learning and memory in a rat model of AD. Furthermore, we investigated the effects of CX691 on BDNF protein expression in the hippocampus tissue.

## Materials and Methods

***Chemical reagents and drugs***

Amyloid-beta (Aβ)1–42 was purchased from Sigma-Aldrich (USA). Before surgery and microinjection, the Aβ1–42 peptide was dissolved in a phosphate-buffered saline solution (PBS) at a concentration of 4 mg/ml and then incubated at 37°C for 72 hr to induce aggregation. CX691 was obtained from Santa Cruz Biotechnology Inc. (USA) and BDNF ELISA kit was purchased from Zellbio (Germany). 1% dimethyl sulfoxide was used as CX691 vehicle. 

***Animals***

Forty male Wistar rats weighing 250–300 g were obtained from the animal house of Rafsanjan University of Medical Sciences. Rats were maintained in a temperature controlled room (23±2 ^°^C) and kept on a 12 hr light/12 hr dark cycle with water and food available *ad libitum*. Attempt was made to minimize animal suffering during the whole experimental course. All experimental procedures in this investigation were approved by the Ethical Committee of Rafsanjan University of Medical Sciences (Ethical code: IR.RUMS.REC.1395.58) based on the United States NIH Guide for the Care and Use of Laboratory Animals (publication no. 85–23).

***Surgery and induction of Alzheimer’s disease***

Animals were anesthetized with ketamine (50 mg/kg, IP) and xylazine (5 mg/kg, IP) and mounted on a stereotaxic apparatus. The skull was opened and above the injection sites, a small hole was then drilled into the skull via a dental burr. The stereotaxic coordinates for bilateral microinjection into the hippocampus CA1 region were obtained from rat brain atlas of Paxinos and Watson (-3.5 mm posterior from Bregma, ±2.0 mm lateral to the sagittal suture and -2.8 mm to outer surface of skull). Microinjections were performed with a flatted-tipped 30-gauge injection needle. A 5-µl Hamilton syringe connected to polyethylene tubing (PE-10) was used to attach the injector cannula. The injector cannula was lowered into the bilateral hippocampus, and 4 µg/1 µl Aβ1–42 was delivered slowly via a microinjection syringe pump. The injection cannula was kept in place for 10 min to allow the injected solutions and tissue to equilibrate and avoid possible reflux through the needle track. Incisions were ligated with nylon thread. The same surgery procedure was performed in the sham group, except that PBS was injected into the hippocampus. After surgery, animals were placed in warmed cages in a dark room and 7 days allowed to recover with free access to water and food. The animal’s general condition, including food and water intake as well as the body weight, were checked daily after surgery (20).

**Figure 1:**
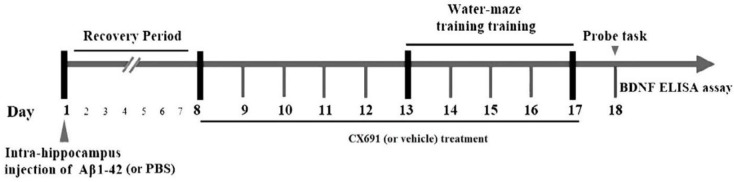
The timeline of the experimental protocol. Animals were divided into 5 groups. In the first day of the experiment period, the amyloid-beta 1-42 (Aβ1-42) or phosphate-buffered saline (PBS) were bilaterally injected into the hippocampus of rats. After 7 days recovery, the animals were treated with CX691 or slain for 10 days (day 8 to 17). Morris water maze (MWM) test was performed on days 13 to 18. In final day of the experiments, animals were killed and their brains were removed for evaluation of brain-derived neurotrophic factor (BDNF) protein expression by ELISA assay

**Figure 2:**
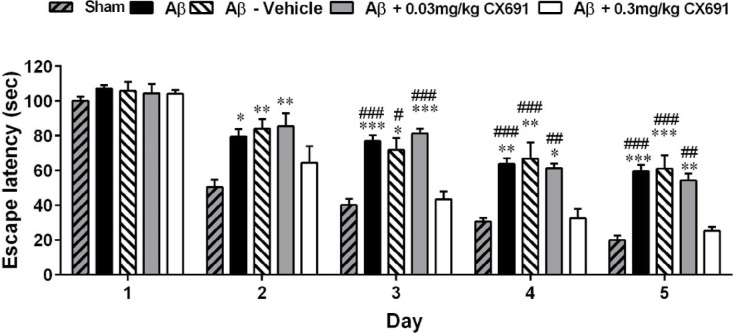
The effect of CX691 treatment on spatial learning function in the amyloid-beta (Aβ)-treated rats. Each block represents the average of escape latency to find the hidden platform in the Morris water maze (MWM) test for five consecutive trial days. Each value is the mean±SEM. n = 8/group. * *P*<0.05, **P<0.01 and*** *P*<0.001 vs. sham group; # *P*<0.05, ## *P*<0.01 and ### *P*<0.001 compared with Aβ + 0.3 mg/kg CX691 group

**Figure 3:**
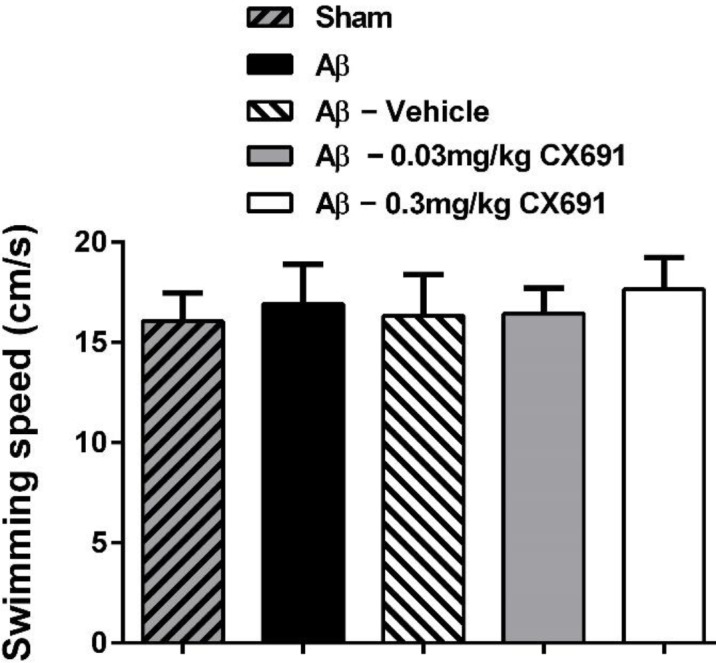
The effect of amyloid-beta 1-42 (β1-42) and CX691 treatment on locomotor function of rats. Each block represents the average of sweeping speed of all experimental groups in day 5 of Morris water maze (MWM) test. Each value is the mean±SEM. n = 8/group

**Figure 4:**
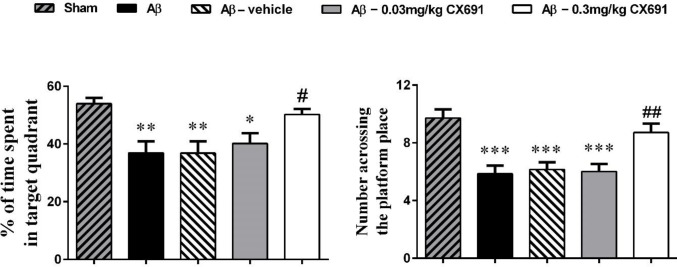
The effect of CX691 treatment on spatial memory function in amyloid-beta (Aβ)-treated rats

The percentage of time spent in the target quadrant (A) and the number of crossing the platform site (B) in the probe task in the Morris water maze (MWM) test. Each value is the mean±SEM. n= 8/group. * *P*<0.05, **P<0.01 and*** *P*<0.001 vs. sham group; # *P*<0.05 and ## *P*<0.01 compared with Aβ and Aβ-vehicle group

**Figure 5:**
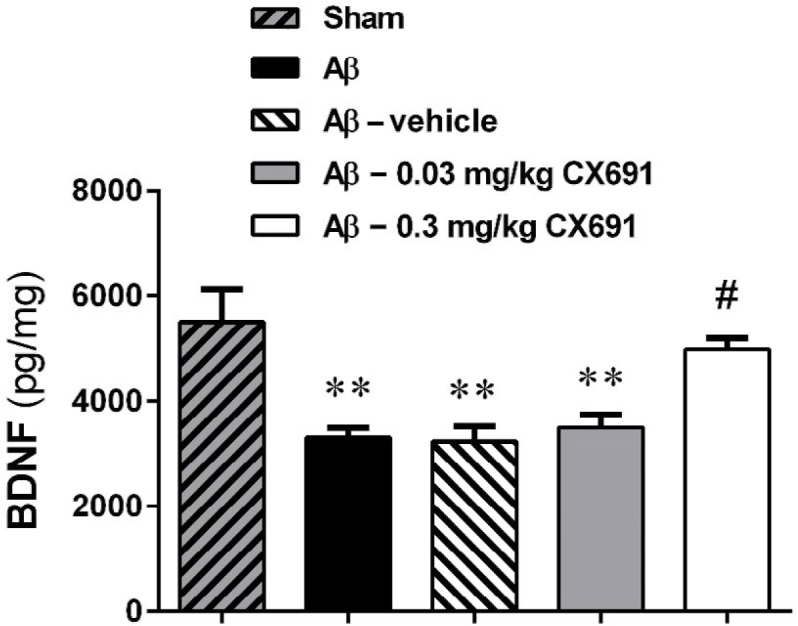
The effects of CX691 on hippocampus brain-derived neurotrophic factor (BDNF) protein expression in the amyloid-beta (Aβ)-treated rats. Relative expression of BDNF was assessed using ELISA method. Each value is the mean±SEM. n=6/group. ** *P*<0.01 compared with sham group

# *P*<0.05 compared with Aβ and Aβ-vehicle group

**Figure 6:**
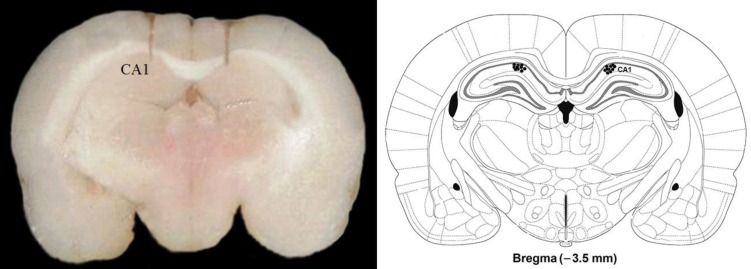
Histological verification of cannula of amyloid-beta (Aβ) injection sites in the CA1 regions of the hippocampus (Left side). Right side of the Figure shows the approximate location of the injection needle tip in the CA1 region (indicated by the black arrows)

***Animal treatment***

The rats were divided into 5 experimental groups (8 animals per group):

(1) Sham group. The animals in this group received intra-hippocampus 1 µl of PBS. 

(2) Aβ-treated (Aβ) group. The animals in this group received intra-hippocampus 4 µg/1 µl ofAβ1–42.

(3) Aβ + CX691-vehicle (Aβ-vehicle) group. The animals in this group received intra-hippocampus 4 µg/1 µl of Aβ1–42 and then treated with CX691-vehicle (two times per day for 10 days; gavaged).

(4) Aβ + 0.03 mg/kg CX691-treated (Aβ-0.03 mg/kg CX691) group. The animals in this group received intra-hippocampus 4 µg/1 µl of Aβ1–42 and then treated with 0.03 mg/kg of the CX691 two times per day for 10 days; gavaged). 

(5) Aβ + 0.3 mg/kg CX691-treated (Aβ-0.03 mg/kg CX691) group. The animals in this group received intra-hippocampus 4 µg/1 µl of Aβ1–42 and then treated with 0.3 mg/kg of the CX691 (two times per day for 10 days; gavaged). 

***Morris Water Maze Test (MWM)***

Spatial learning and memory were assessed using MWM. On the second 5 days of CX691 treatment, the animals performed 4 trials per day for 4 consecutive days. Briefly, the animals were placed into the water facing the water pool wall from 4 different quadrant sites. In this part of assessment, the values of escape latency and swimming speed of animals were recorded. The time for each test was 120 sec. If the rats did not find the escape platform within 120 sec, they were directed to the platform to stay there for 15 sec. The probe test was performed 24 hr after the last trial of place navigation. In the probe test, escape platform was removed, and the animals were placed into the water at any selected point of pool quadrants facing the tank wall. The values of swim time in the target quadrant (place of removed platform) and the number of the platform-site crossovers were measured (21).

***Assessment of BDNF protein expression***

After the completion of MWM assessment, animals were sacrificed under deep anesthesia. Hippocampi were rapidly dissected on ice, immediately frozen in liquid nitrogen and stored at -80 ^°^C. The level of BDNF in each hippocampus was measured using rat BDNF ELISA kits (ZellBio, Germany). Hippocampus was cut into small pieces and homogenized in PBS on ice. The homogenates were then centrifuged for 15 min at 6000 rpm at 4 ^°^C. The supernatant was assayed in strict accordance with the instructions in the ELISA kit.

***Histological verification***

Histological verification of the injection sites was carried out in 10 animals that were randomly chosen; two from each of the five experimental groups. After the end of behavioral assessments, those animals were killed. The rat’s brains were removed and fixed in 10% formalin solution. Brain sections were examined to determine of the injection place. The injection placements were confirmed using rat brain atlas of Paxinos and Watson.

***Statistics***

The data were presented as mean±SEM. Statistical analysis was performed using one- or two-way ANOVA followed by Tukey’s *Post hoc* test. Statistical significance was considered when *P*<0.05.

## Results

***The effects of CX691 on cognitive function in amyloid beta 1-42 treated animals***

The protocol of our experiments is shown Figure 1. The animals were randomly separated into two main groups for stereotaxic surgery: 1) PBS-operated group (sham) and 2) Alzheimer (Aβ1–42 treatment) group (these groups of rats were divided into 4 groups: Aβ, Aβ + vehicle, Aβ + 0.03 and Aβ +0.03 mg/kg of CX691). The rats in the Alzheimer group received bilateral intra-hippocampus administration of Aβ1–42. The MWM test was used to evaluate the ability of learning and process spatial information of rats. 45 min before the water-maze training, CX691 (at 0.03 and 0.3 mg/kg doses / or vehicle) were administered to the animals. The rats swimming time and distance with various CX691 doses treatments after five days of MWM training are presented in Figure 2-5. As expected, the Aβ and Aβ + vehicle-treated rats showed a significant delayed escape latency after 5 days of water-maze training indicating the impairment of learning in these animals compared to the sham group (*P*<0.001). Two-way ANOVA revealed significant effects of 0.3 mg/kg CX691 treatments on escape latency of Aβ1–42 treated animals (*P*<0.001) (Figure 2). No difference was found between the Aβ + 0.3 mg/kg CX691-treated and the sham rats. This data revealed that CX691 could enhance the spatial learning performance defects in Aβ--treated animals. It is notable that 0.03 mg/kg administration of CX691 for 10 days cannot decrease the escape latency in Aβ1-42-treated animals (*P*>0.05) (Figure 2). Neither the training nor the treatments have important effects on the rats swimming speed in day 5 of MWM test, representing that those animals do not show any locomotor deficits (*P*>0.05) (Figure 3). Administration of 0.3 mg/kg CX691 significantly prevented the Aβ1-42-induced decrease in both time spent in the right quadrant in the MWM probe task (*P*<0.05) (Figure 5A) and attempts for exploring the escape platform (*P*<0.01) (Figure 4B). In non-target quadrants, no significant difference was observed among different experimental groups. 

***The effects of CX691 on BDNF protein levels in amyloid beta 1-42-treated rats***

Expression of hippocampus BDNF protein was measured via ELISA detection method. As shown in Figure 5, administration of Aβ significantly decreased the BDNF protein level compared to the sham group (*P*<0.01). According to the obtained results, the BDNF protein level within the hippocampus tissue was significantly elevated in Aβ-injected animals treated with 0.3 mg/kg CX691 (*P*<0.05). It is noteworthy that 0.03 mg/kg administration of CX691 for 10 days did not increase the hippocampal BDNF protein level in Aβ-treated rats (*P*>0.05) (Figure 5).

***Histology***

Figure 6 at right side presents the photomicrograph of needle trace for hippocampus CA1 area microinjection of Aβ or vehicle. Left side of Figure 6 shows the coronal section schematic illustration, which was taken from the Paxinos and Watson rat brain atlas (Figure 6).

## Discussion

In present study, the effects of CX691 on memory function and expression of BDNF were investigated in a rat model of AD.

We observed that injection of Aβ1-42 into the hippocampus causes impairment of rat performance in MWM as a task known to involve spatial memory. Our data showed that treatment with CX691, as a positive modulator of AMPA receptors, attenuates Aβ-induced impairment in water maze task performance. Additionally, our findings revealed that the direct injection of Aβ1–42 into hippocampus results in a reduced BDNF protein level in this tissue. On the other hand, our data showed that CX691 administration can elevate BDNF protein level in hippocampus tissue of Aβ1–42-treated rats. 

The accumulation of Aβaggregation in the brain in AD leads to the progressive synapses disruption and neuronal networks (22) cognitive decline, and devastating neurodegeneration, not only as a result of the extracellular accumulation of beta-amyloid peptide (Aβ. Several prior investigations have shown that increased Aβ reduces the excitatory synaptic transmission through reducing the amount of surface AMPA and NMDA receptors, which is in turn associated with a collapse of glutamatergic dendritic spines (23–25).

As reviewed in the introduction of this manuscript, cognitive decline in AD is highly correlated with loss of synapse in AD human brain and animal AD models and reduced AMPA receptor-mediated synaptic transmission in Alzheimer situation (3–5) genetic, animal model, and biochemical studies has indicated that the accumulation of amyloid-beta (Aβ).

Ampakines act as a positive allosteric modulators of AMPA-type glutamate receptors (26, 27). In experimental animal models, the ampakines have been shown to facilitate hippocampal LTP, a mechanism related with formation and storage of memory (27–29). Animal studies have also revealed that ampakines could improve the performance in a variety of memory tasks such as spatial mazes (30), discrimination of odor (31), eye-blink conditioning (32), a spatial delayed-non-match-to-sample task (33), and learned fear (34). It has been shown that ampakines could be effective in reducing age- associated memory impairment in rats (30). 

On the other hand, BDNF contributes to synaptic plasticity and is protective in animal models of neurodegenerative diseases and brain injury (35,36).

Several studies have demonstrated that Aβ administration can result in the reduction of brain and hippocampus BDNF level. It has been shown that central administration of Aβ1-42 reduces the serum and brain BDNF level in animal models of AD (21, 37). 

It is notable that some previous studies have provided evidence supporting the idea that neurotrophic factors could be promising drug candidates for the treatment of AD and other neurodegenerative disorders (21,38–40). In this regard, they indicated that BDNF can prevent the neuronal loss in the hippocampal formation, basal forebrain and cortex of injured adults (41, 42). Thus, reduced BDNF levels can contribute to the synaptic loss and atrophy of neurite observed in the AD patients’ brains. Furthermore, BDNF upregulation could control the progress of AD and improve the cognitive function (38, 39). 

The positive modulators of AMPA receptors have also been proposed to raise BDNF, (43), which involved in neurogenesis, neuronal survival/stability and neuroplastic procedures (44). Thus, an additional way by which positive modulators of AMPA receptor possibly induce central neuroplastic changes and, finally, cognitive improvement is through prompting the BDNF release (28). The aforementioned modulators have been shown to increase BDNF mRNA and protein expression in cultured cortical (45, 46) and hippocampal (47) neurons as well as following acute and sub-chronic administration *in vivo*. 

Woolley and colleagues investigated the CX691 effect in three animal models of learning and memory, neurochemistry in the medial prefrontal cortex and dorsal hippocampus after acute administration, and on BDNF mRNA expression in the hippocampus of rats following sub-chronic and acute administration. Their findings revealed that CX691 attenuates scopolamine-induced cued-fear conditioning impairment after acute treatment and a temporally-induced deficit in novel object recognition after both acute and sub-chronic treatment. It also improved attentional set-motion after sub-chronic administration. In addition, they showed that acute CX691 administration increases extracellular levels of acetylcholine in medial prefrontal cortex and dorsal hippocampus and dopamine in the medial prefrontal cortex CX691. Sub-chronic administration has been found to raise the expression of BDNF mRNA in both the whole hippocampus and CA1 sub-region (48).

## Conclusion

In summary, CX691 (as a positive modulator of AMPA receptors), exhibited memory enhancement efficacy in Aβ 1-42 rat model of AD. This pro-cognitive property was further supported by elevated levels of BDNF protein, a neurotrophic factor involved in neuronal plasticity and neurogenesis in the hippocampus tissue. Altogether, these findings support the pro-cognitive activity reported for CX691 and propose that this agent might be beneficial in managing and controlling the progression of AD.
